# What Does a Hospital Survey on Patient Safety Reveal About Patient Safety Culture of Surgical Units Compared With That of Other Units?

**DOI:** 10.1097/MD.0000000000001074

**Published:** 2015-07-13

**Authors:** Qin Shu, Miao Cai, Hong-bing Tao, Zhao-hui Cheng, Jing Chen, Yin-huan Hu, Gang Li

**Affiliations:** From the Department of Health Administration (QS, MC, HT, ZC, JC, YH), School of Medicine and Health Management, Tongji Medical College, Huazhong University of Science and Technology; and Tongji Hospital (GL), Tongji Medical college, Huazhong University of Science and Technology, Wuhan, Hubei Province, P.R. China.

## Abstract

The objective of this study was to examine the strengths and weaknesses of surgical units as compared with other units, and to provide an opportunity to improve patient safety culture in surgical settings by suggesting targeted actions using Hospital Survey on Patient Safety Culture (HSOPSC) investigation.

A Hospital Survey on Patient Safety questionnaire was conducted to physicians and nurses in a tertiary hospital in Shandong China. 12 patient safety culture dimensions and 2 outcome variables were measured.

A total of 23.5% of respondents came from surgical units, and 76.5% worked in other units. The “overall perceptions of safety” (48.1% vs 40.4%, *P* < 0.001) and “frequency of events reported” (63.7% vs 60.7%, *P* = 0.001) of surgical units were higher than those of other units. However, the communication openness (38.7% vs 42.5%, *P* < 0.001) of surgical units was lower than in other units. Medical workers in surgical units reported more events than those in other units, and more respondents in the surgical units assess “patient safety grade” to be good/excellent. Three dimensions were considered as strengths, whereas 5 other dimensions were considered to be weaknesses in surgical units. Six dimensions have potential to aid in improving events reporting and patient safety grade. Appropriate working times will also contribute to ensuring patient safety. Medical staff with longer years of experience reported more events.

Surgical units outperform the nonsurgical ones in overall perception of safety and the number of events reported but underperform in the openness of communication. Four strategies, namely deepening the understanding about patient safety of supervisors, narrowing the communication gap within and across clinical units, recruiting more workers, and employing the event reporting system and building a nonpunitive culture, are recommended to improve patient safety in surgical units in the context of 1 hospital.

## INTRODUCTION

Patient safety is one of the most urgent issues in any health care system, and this issue has received increasing attention in China. The Institute of Medicine (IOM) declared that hospitals must build a patient safety culture in order to enhance patient safety.^1^

A positive safety culture will improve a hospital's patient safety performance, which could help the organization strengthen its safety outcomes. Moreover, a negative safety culture will encourage the hospital to address issues in patient safety management. Pronovost^2^ stated that the first thing to accomplish in improving patient safety in a hospital is to assess the patient safety culture. In this study, for the Hospital Survey on Patient Safety Culture (HSOPSC)^3^ has been widely used and can be tested on units’ level, we selected it as the measurement tool to assess the patient safety culture of hospital units. HSOPSC was first introduced by the Agency of Healthcare Research and Quality (AHRQ) in America. Significant work still needs to be done in the sampled organization and in the context of the region in general to improve patient safety practices and culture.^4^

A growing number of studies on unit-level assessment have emerged. Intensive care units (ICUs) have been the most common subjects of studies, such as Ballangrud,^5^ who indicated that fields where improvements are needed in the ICUs include incident reporting, feedback and communication about errors, and organizational learning. Kho^6^ highlighted the importance of teamwork across units in ensuring a positive safety culture. Many other scholars^5–7^ have used various measurement tools and different views in their studies, achieving unique results. Scherer^7^ compared the perceptions of physicians and nurses in the perioperative area, and suggested the safety culture dimensions of “supervisor/manager expectations and actions promoting safety” and “feedback and communication about error” had significant room for improvement. Hoffmann^8^ conducted an open randomized controlled trial and evaluated the effects on patient safety culture in general practice. Other surveys also assessed general practice units.^9^

However, few studies have focused on surgical units. Surgical units consist of highly advanced equipment, caregivers with varying levels of expertise, vulnerable patients, and very limited time.^10^ Furthermore, surgical units are complex and have high potential hazard for patient harm and adverse events.^11^ More than half of all adverse events (51%–62%) occur in surgical settings.^12^ Promoting highly reliable care in surgical environments requires a strong patient safety culture.^13^ Therefore, patient safety culture in surgical units is an urgent concern. An even lesser number of studies measure surgical settings using HSOPSC. A similar study was conducted by Kaafarani^14^ 5 years ago, but he used another tool and his findings indicated an inadequate strategy for culture building. Few studies have assessed patient safety culture in Chinese hospitals.^4^ Different hospitals have different patient safety cultures in surgical units. Thus, the objective of this study was to examine the strengths and weaknesses of surgical units compared with other units, and to improve patient safety culture in surgical settings by suggesting targeted actions at the hospital unit level by using HSOPSC investigation.

## METHODS

### Design

The design used for this study was a cross-sectional survey.

### Setting and Sample

All nurses and doctors working at the Affiliated Hospital of Jining Medical College were included in the study. The hospital is a tertiary general hospital located in southwest Shandong, China, and is the largest teaching hospital in Jining, Shandong. The hospital has 3000 beds and 2943 health care workers (including 558 in surgical units and 2385 in other units), and served 1,867,181 outpatient customers and 119,722 inpatient customers in 2013. The annual number of surgical procedures performed in the hospital exceeded 79,267 in the same year.

All surgical departments and other departments were included, with a total of 2230 individuals participating in the survey. The 713 remaining personnel were not included in the study because of illness, vacation, working in other places, training, or their unwillingness to participate.

### Instrument

The investigation instrument was a validated Chinese version of the HSOPSC.^15^ The Chinese version of the HSOPSC was previously validated for paper distribution. To make the participants understand it, the questionnaire was translated into Chinese before giving out to the respondents. Translations were conducted by 2 independent researcher groups. Readability and functionality of the questionnaire was pilot-tested on several health care workers and research personnel to ensure that the concepts were correctly worded and conceptualized. After making the pilot test, the authors determined that the order of the questionnaire did not suit the habits of the Chinese and that 2 result items might be overlooked by respondents, so we adjusted the order of some questions.

HSOPSC was developed by the Agency for Healthcare Research and Quality in 2004 as a safety culture assessment tool. The HSOPSC includes 12 dimensions (a total of 42 items) that indicate the perceptions of patient safety culture. Each dimension contains 3 or 4 items (Table 2). Every item is measured by a 5-point Likert scale. Patient safety grade was also scored from 1 to 5 as follows: (1) excellent, (2) very good, (3) acceptable, (4) poor, and (5) failing. The number of adverse events reported by the respondent during the last 12 months is scored from 1 to 6: (1) no report, (2) 1 to 2 reports, (3) 3 to 5 reports, (4) 6 to 10 reports, (5) 11 to 20 reports, and (6) >21 reports.

**TABLE 2:**
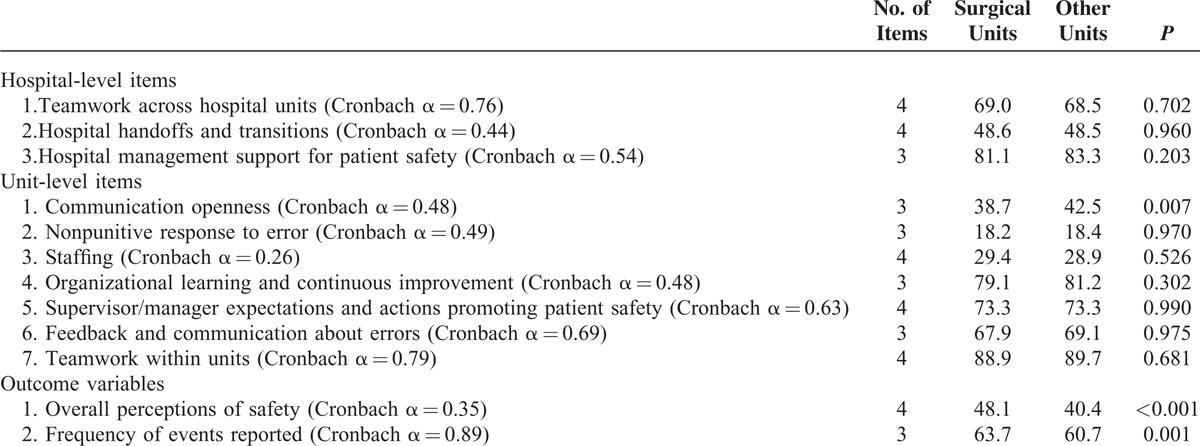
Safety Culture Dimension Percent Positive Scores Between Units

Respondents’ characteristics, including profession, years in the hospital, years of working on the units, years of working as the current profession, working hours per week, and whether the respondent was in contact with patients, were also included. Adjustments such as placing characteristics at the front of the questionnaire, and placing patient safety grade and number of events reported (NER) at the end of the questionnaire, were made to suit the habits of Chinese respondents and reduce the missing rate in the responses.

### Data Collection

The investigation was conducted in July 2014.

We sent questionnaires to the medical personnel through the online hospital e-mail system. The timeline was 1 month, and medical personnel would receive e-mail reminders every week until they completed the questionnaire.

### Statistical Analysis

Statistical Product and Service Solutions (SPSS) 12.0 was used to analyze the data. Descriptive statistics was used to describe the frequency and percentages of the samples. Negatively worded items were reversed to ensure that positive answers indicated a high score. Positive response means the answers “excellent” or “very good” in positively worded items. The positive response rates between the 2 units groups and between physicians and nurses in each unit group were compared on every dimension. Pearson χ^2^ tests were used to compare the positive proportion of “number of incidents reported,” and “patient safety grade” between the surgical units and other units. The outcome on patient safety grade was recorded in 3 categories, including “poor or failing,” “acceptable,” and “excellent/good.” The outcome on the NER was recorded into “>5 events,” “3 to 5 events,” “1 to 2 events,” and “no events.”^4^ A multiple regression analysis was conducted using overall patient safety grade, the NER was taken as dependent variables, and controlling for every dimension and every general characteristic worked as independent variables. The 2 categorical outcomes were then adopted for ordinal logistic regression. Internal consistency of the factors was assessed using Cronbach α. The conventional level of *P* ≤ 0.05 was taken to represent statistical significance.

### Ethics

The respondents voluntarily participated in the survey. A consultant in the hospital research department administered the web-based questionnaire with informed consent and the returned questionnaires were without identifying marks. The authors promised that ethical issues such as data fabrication, double publication, plagiarism, and so on were forbidden in the article.

## RESULT

### Sample

A total of 2230 participants, 525 (23.5%) of them were from surgical units and 1705 (76.5%) from other units, completed the questionnaires. The overall response rate was 75.7%, including 94.1% in surgical units and 71.5% in other units. The surgical unit groups were referred to the units conducting surgical removal or repair, and the other units were referred to the rest units of the hospital including most internal medicine units and other units. Table 1 shows details on the characteristics of the respondents.

**TABLE 1:**
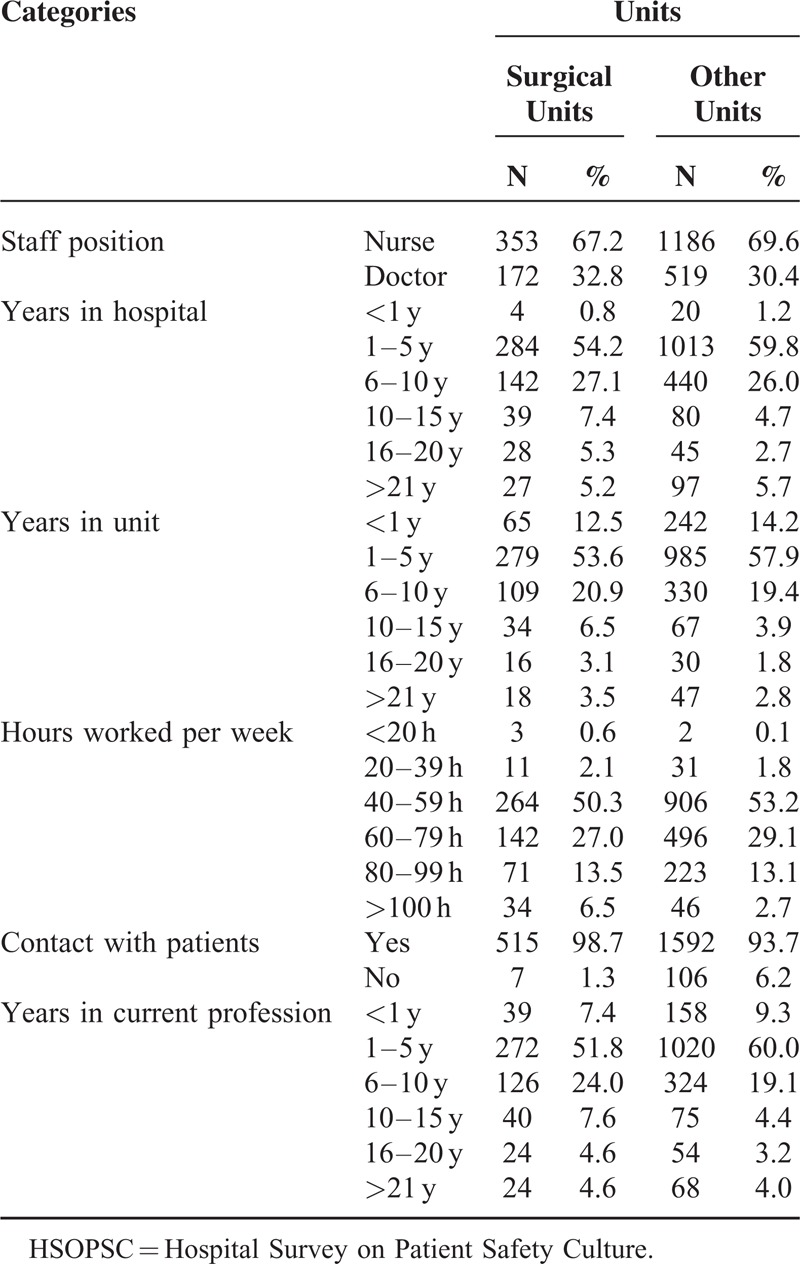
Characteristics of 2230 Respondents Based on HSOPSC Results

### Strengths and Weaknesses of Surgical Units Compared With Other Units

The percent positive ratings (PPRs) on the dimensions at the hospital level were between 29.1% and 89.7%, between 18.2% and 88.9% at the surgical unit level, and between 17.1% and 89.7 % at other unit level (Table 2).

Among the 12 patient safety culture dimensions, dimensions that received PPR exceeding 75% are considered as areas of strength^4^; these dimensions include “teamwork within units” (88.9%), “hospital management support for patient safety” (81.3%), and “organizational learning and continuous improvement” (79.1%), which had the 3 highest PPRs. On the contrary, the dimensions with PPRs <50% can be considered as fields of weakness and include “communication openness” (38.7%), “overall perceptions of safety” (37.1%), “hospital handoffs and transitions” (36.3%), “staffing” (29.4%), and “nonpunitive response to error” (18.2%).

The difference between surgical units and other units were identified, as shown in Table 2. Three dimensions were found to be different: the “communication openness” of surgical units was weaker than that of other units (38.7% vs 42.5%, *P* = 0.007), whereas the “overall perceptions of safety” (48.1% vs 40.4%, *P* < 0.001) and “frequency of events reported” (63.7% vs 60.7%, *P* = 0.001) of surgical units performed better than other units. The comparison of other dimensions indicated statistically similar PPRs between the surgical units and other units.

The items considered as dimensions of strengths and weaknesses were examined further.

The results showed that in the “communication openness” dimension, 1 major item of strength was highlighted by the responses to the item on “Staff is afraid to ask questions when something seems not right.” For this item, in respondents’ perception, other units performed much better than surgical units (66.8% vs 32.8%, *P* < 0.001).

The items “overall perceptions of safety” and “frequency of events reported” were also analyzed. The results demonstrated that surgical units had less “patient safety problems” than other units (31.4% vs 23.4%, *P* < 0.001). Surgical units reported more frequent mistakes than other units. The item “When a mistake has been made, but it will not harm the patient, how often is this reported?” (64.5% vs 58.5%, *P* = 0.016) showed significant contributions on the outcome item “frequency of events reported.”

### Comparisons Between Units in Patient Safety Grade and the Number of Events Reported

Most of the sampled respondents indicated “good” or “excellent” hospital patient safety grades (82.6% and 75.3%, respectively, in surgical units and other units) (Table 3). Nearly half of the sampled respondents reported no events (48.0% and 52.2%, respectively), whereas 6.0% and 5.0% of the respondents reported >5 events, more detailed information shown in Table 3.

**TABLE 3:**
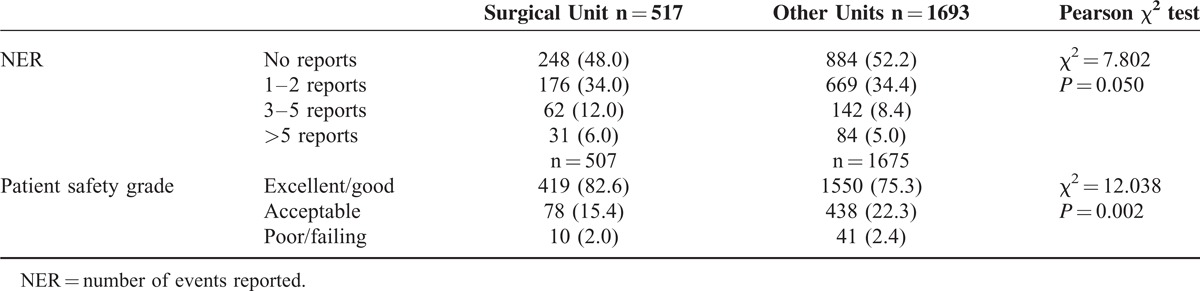
Comparisons Between the 2 Unit Groups Regarding the Number of Events and Patient Safety Grade

Statistically significant differences between the 2 types of units were observed. For instance, more medical incidences were reported in the surgical units than in the other units. A significantly higher proportion of respondents in the surgical units also took the patient safety grade as “excellent/good” (Table 3).

### Factors Influencing Patient Safety Grade and Events Reported in Surgical Units

For “patient safety grade,” participants with 80 to 99 working hours (odds ratio [OR] = 3.79) and those with 40 to 59 hours (OR = 4.67) reported better patient safety grade than those with >100 working hours. Participants who scored higher on communication openness, teamwork within units, supervisor expectations and actions promoting patient safety, feedback and communication about error, and teamwork across hospital units also rated higher patient safety grades.

Teamwork across hospital units was a protective factor on “NER.” Participants with 10 to 15 years of hospital experience reported fewer events than those with >21 years of experience (OR = 7.49). Table 4 shows more details.

**TABLE 4:**
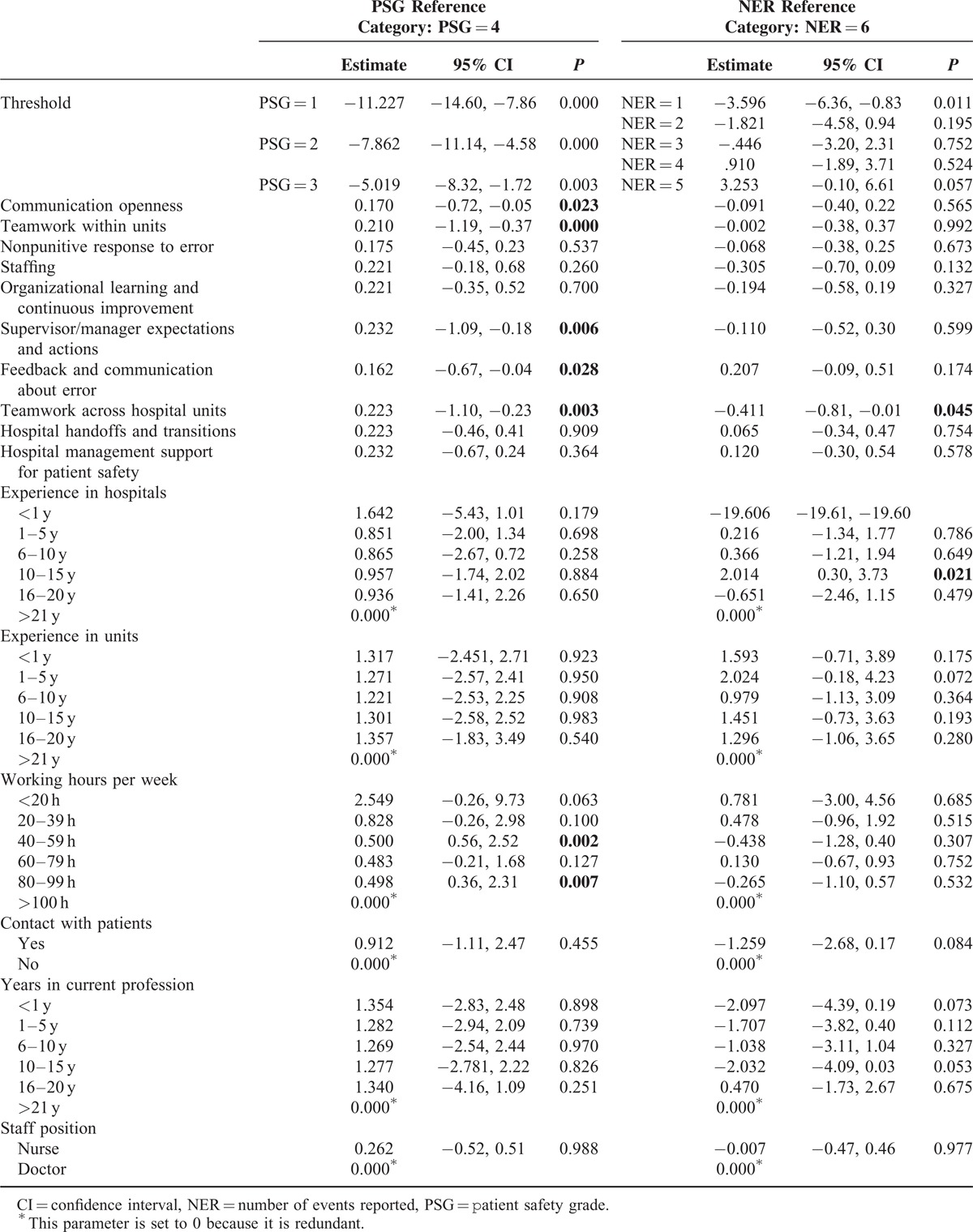
Ordinal Logistic Regression Model

### Factors That Influence the Overall Perception of Safety and Frequency of Events Reported in Surgical Units

We conducted multiple regressions to determine the factors that influence patient safety and events reported at the surgical unit level. The results showed that several dimensions can affect 2 outcome dimensions, whereas the general characteristics had no correlation with the outcome dimensions.

### Overall Perception of Safety

Linear regression analysis demonstrated that a 1-unit increase in the dimensions of “supervisor/manager expectations and actions” and “teamwork across hospital units” was found to increase overall perception of safety score by 0.11 (*P* = 0.020) and 0.13 (*P* = 0.011), respectively.

### Frequency of Events Reported

Three dimensions were found to affect the frequency of events reported. Frequency of events reported improved by 0.23(*P* < 0.001) for a 1-unit increase in the score on communication openness, by 0.19 (*P* = 0.013) for every unit of increase in the score on supervisor/manager expectations and actions, by 0.17 (*P* = 0.029) for a unit increase in the score on hospital handoffs and transitions, and by 0.36 (*P* < 0.001) per unit increase in the score on feedback and communication about error.

## DISCUSSION

This is one of the fewest units’ studies evaluating patient safety culture in a Chinese hospital. The results showed that “overall perceptions of safety” in surgical units was better than that in other units, and “frequency of events reported” performed a little better in surgical units. Medical workers in surgical units reported more events than those in other units, and more respondents in the surgical units reported a good/excellent patient safety grade. On the contrary, the communication openness of surgical units performed worse than other units, although other similar studies did not find difference comparing the surgical units with other units.^14^

Dimensions “communication openness” and “feedback and communication about error” were associated with higher patient safety grade and more frequency of events reported. Dimension “teamwork across hospital units” was associated with more NER, higher patient safety grade, and higher overall perception of safety score. Dimension “supervisor expectations and actions” were associated with higher patient safety grade, overall perception of safety score, and more frequency of events reported higher. Dimension “hospital handoffs and transitions” was associated with more frequency of events reported. In this article, it is assumed that higher frequency of events reported is associated with better patient safety culture. The positive association seems to be a confliction, but actually it is not. Physicians and nurses all over the world are likely to underreport patient safety events due to a number of reasons, the number is normally much less than the actual number. It is wiser to report the case and summarize the reasons of making mistakes when an error occurs, rather than punishing the error maker. These reports will be used to make root cause analysis and prevent similar errors. Therefore, it is believed that in a Chinese hospital context, the more events reported, the more emphasis medical professionals put on patients’ safety, and the higher patient safety grade is.

Our study also indicated that “teamwork within units,” “hospital management support for patient safety’,’ and “organizational learning and continuous improvement” were the top 3 items of surgical unit respondents. The results also highlighted 5 items to be improved, namely “communication openness,” “hospital handoffs and transitions,” “staffing,” “overall perceptions of safety,” and “nonpunitive response to error.” “Teamwork within units” and “organizational learning and continuous improvement” have been proven by numerous domestic^16,17^ and international^18,19^ studies to be strengths. However, the dimension “hospital management support for patient safety” was not always considered as strength.^20^

The first dimension to be improved is “communication openness.” “Communication openness” has always been considered to be a moderate dimension by most studies^21^ or what performs well in general practice units.^9^ However, the dimension was considered as a weakness in surgical units in our study. More importantly, our results show that “communication openness” might improve the patient safety grade and frequency of events reported in surgical units. Furthermore, this aspect was 1 of the 3 significant differences found among the 12 dimensions through comparisons between the 2 unit groups. Surgical units performed worse than other units, especially in the dimension “staffs are afraid to ask questions when something do not seem right.” In surgical units, one can argue that complicated operations that involve difficult methods and unexpected complications can only be addressed by few physicians. The physicians who have sophisticated knowledge of technology were unlikely to teach others because an exclusivity of their techniques ensures their authority and the monopoly of the income. These individuals were always considered to be right, and junior doctors have difficulty in expressing their criticism to the authority for cultural reasons. This is not likely to be beneficial for improving patient safety care because potential errors made by the authorities may be ignored and cause potential harm to patients. Therefore, the new staff should have equal chance to speak freely and be encouraged to ask questions about the problems when something seems not right. Atmosphere with open communication has been highly recommended by various researchers.^28,29^

The next problem that needs to be addressed is the nonpunitive response to events. Our study demonstrated that nearly half of the respondents did not report any event in the past 12 months, which is a major concern for both surgical and other units. This dimension “nonpunitive response to error” got one of the lowest ratings, which was consistent with other studies.^20,22^ The majority of respondents felt their mistakes were being held against them and were later recorded in their personnel files (a lifelong document which records the professional experience of employees). A study conducted by Evans^23^ revealed that a punitive response to error is a major barrier that prevents error reporting between doctors and nurses.^22^ The US experience can shed light on our practice. In 1980s, the peer-review process which aimed to ensure medical safety was converted into a punitive process after the famous multimillion-dollar lawsuit.^24^ The Chinese personnel files actually play the role of Data Bank Records and “have economic impact on physicians” as Livingston commented.^24^ In Chinese hospitals, the reporting of medical errors often brings trouble to the fame and salary of health care professionals, although the reporting systems are claimed as nonpunitive.^25^ A nonpunitive culture should therefore be developed. Hospital should consider abolishing potential rules and regulations on punitive response for minor medical errors while establishing a mandatory reporting system for those major errors.^1^

Several studies have proved that a lack of feedback about errors can impede incident reporting.^26,27^ Weaver^28^ defined an environment with specific feedback and communication about errors, and made proactive discussions regarding how patient could benefit from a supportive, learning-oriented patient safety culture. Staff members with longer years of experience in hospitals understood the importance of medical quality and event reporting and therefore reported more events, and there is a finding that agreed with Evans’ study.^23^ Hence, the above aspects should be taken into consideration to enhance the reporting of events in surgical units.

Another finding was that teamwork across hospital units, as well as supervisor expectations and actions, positively influenced both the overall perception of safety and patient safety grade. Our results demonstrated that teamwork across hospital units obtained high PPRs for overall perception of safety, which was higher than those of other hospitals in China,^16^ and higher than those of ICUs^5^ in other countries. This aspect should be maintained and enhanced to guarantee good patient safety culture. Supervisor expectations and actions may have a direct impact on staff consciousness: the more supervisors emphasize on patient safety, the more importance the staff puts on it. Therefore, it is important to draw the attention of managers to improve patient safety culture.

“Hospital handoffs and transitions” is a factor with a low score, and has been significantly associated with a higher frequency of events reported. This dimension has often been reported in other studies, but was not classified as a weakness; however, it had low PPRs in both surgical units and other units. For example, Snijders^29^ reported that the PPR of hospital handoffs and transitions in neonatal ICUs was >60%. In the same surgical setting, Haugen^11^ did not take hospital handoffs and transitions as weakness either. However, 1 study considered the dimension to affect the patient safety culture in Lebanese hospitals.^22^ Many respondents admitted that major problems occur in the exchange of information across units and the transfer of patients. Hence, “hospital handoffs and transitions” is critical in a health care environment because the consequence is usually significant, such as the loss of important patient care information. Not only does the dimension delay the treatment of patients, but also triggers medical disputes. Evidence has shown that the item “handoffs and transitions” is an important contributor to adverse events, and our results showed that there were a certain positive relationship between hospital handoffs and the frequency of events reported. We therefore recommend that every step of a transfer be traced to identify the key link and make necessary changes to the communication system. For the communication issue, a standard communication procedure during handoffs and transitions should be moved up to the agenda of managers.

Staffing is another problem in patient safety culture. In the results, the PPRs of both surgical and nonsurgical groups concerning staff dimension were very low. In fact, most hospitals all over the world are faced with this problem, but the problem is accentuated in China. Issues on “staffing” indicated that most of the respondents felt that staff allocation was not adequate to handle patient safety-related workload.^30^ Nearly 98% of the health workers worked for >40 h/wk, and half of the respondents worked for >60 h/wk. Most health workers overloaded with work suffer from stress and anxiety, which increase the frequency of adverse events,^22^ and may result in patient safety problems in the surgical units. Moreover, the staff who worked 40 to 59 hours and 80 to 99 hours a week provided better patient safety grade assessment than those who worked for >100 h/wk. Therefore, recruiting more health care workers is highly recommended in the Chinese health care context.

## LIMITATIONS

Our study has several limitations. Although the authors exerted their best efforts at collecting questionnaires, several respondents cannot fill the questionnaire. In addition, we selected only 1 investigation tool to measure the patient safety culture, and hence the possibility of such biases cannot be completely dismissed.

The Cronbach α values were low (ranging from 0.26 to 0.89). The HSOPSC user's guide indicates that a value ≥0.6 is acceptable,^15^ whereas El-Jardali's^4^ Cronbach α values were as low as 0.21. The reason behind the low Cronbach α values could possibly be due to the cultural differences between China and other countries. Different expressions may deliver different information. Thus, we decided not to delete items with low consistency in order to compare our results with those of other studies. Despite these limitations, the methodology and ideas of our survey may be helpful for those interested in measuring baseline survey culture in hospitals.

## CONCLUSION

Surgical units may represent a different culture than other hospital units because of the specific skills and disciplinary traditions on the unit.^5^ Much can be done in the context of 1 hospital to improve patient safety in surgical units in the investigation. Narrowing the communication gap within clinical units, or giving an equal chance to everybody to speak freely about the patient safety, was recommended. Standard procedure on the hospital handoffs and transitions to reduce the errors should be moved up to the agenda. The supervisor should also attach priority to the patient safety and play a key role in promoting the culture. In addition, recruiting more workers, using the reporting system as frequently as possible, and building a nonpunitive culture were also recommended to ensure more patient safety.
